# Supplementary material for: Mercury and Prenatal Growth: A Systematic Review
Source: Int J Environ Res Public Health. 2021 Jul 3;18(13):7140. doi: 10.3390/ijerph18137140 (PMC8297189; doi:10.3390/ijerph18137140)
Supplement: Supplementary file 1 [file ijerph-18-07140-s001.zip › ijerph-1264975-supplementary.pdf]

International Journal of
Environmental Research and Public Health

MDPI

Supplementary File

Part 1: Changes to the protocol.
Part 2: Search terms.
Part 3: Fields used in data extraction.
Part 4. Quality assessment tool.
Part 5. Mercury exposure characteristics in included studies.
Part 6: Results reported in included studies.
Part 7. Data used in albatross plots.
Part 8. Results of NIH Quality Assessment.
Part 9. Results reported in high quality studies and albatross plots.
Part 10. Publication bias analysis.

Part 1: Changes to the protocol.

Change	Rationale
Divided review into two projects to be published separately: 1. Mercury and fetal growth. 2. Mercury and neurodevelopment.	More studies were found than expected, and if all outcomes were discussed in a single paper it would not be possible to adequately discuss all the papers found.
Additional criteria:<br>Include: Studies that used multivariable statistical methods.<br>Exclude: Studies that used only univariable methods such as correlation, *t*-test, etc.	A wide range of potential factors can confound the relationship between mercury and fetal growth. The results of univariable analyses could not answer the research question of this review in a meaningful way.
Additional criteria:<br>Exclude: Studies of Ethylmercury (etHg).	Exposure to ethylmercury is primarily through vaccines, and this has been thoroughly studied elsewhere.
Added technical details of mercury measurement (method, LoD) to data extraction.	Mean mercury concentrations differed significantly between studies, and these additional details may help understand why.
Replaced the Newcastle Ottawa Scale with NIH QA Tool for Observational Cohort and Cross-Sectional Studies.	The NOS scale was not suitable for many of the included studies. The NIH QA Tool proved to be more sensitive and could be applied to both cohort and cross-sectional studies.
Did not meta-analyse study results.	This was not possible because of the wide range of exposures and types of model estimate reported.

Part 2: Search Terms

The following search strategy was adapted to each database.

Population		Exposure		Outcome		Filter
Fetal	AND	Mercury	AND	birthweight	NOT	Animals
Prenatal		Methylmercury		birth weight		
Pre-natal				birth length		
Gestation				head circumference		
Post-natal						
Postnatal						
Neonatal						
Infant						
Infancy						
Baby						
Pregnancy						
Maternal						

<u>Pubmed</u>

("fetal"[Title/Abstract] OR "prenatal"[Title/Abstract] OR "pre-natal"[Title/Abstract] OR "gestation"[Title/Abstract] OR "post-natal"[Title/Abstract] OR "postnatal"[Title/Abstract] OR "neonatal"[Title/Abstract] OR "infant"[Title/Abstract] OR "infancy"[Title/Abstract] OR "baby"[Title/Abstract] OR "pregnancy"[Title/Abstract] OR "maternal"[Title/Abstract]) AND ("methylmercury compounds"[MeSH Terms] OR "mercury"[MeSH Terms]) AND ("birth weight"[MeSH Terms] OR "birth length"[Title/Abstract] OR "head circumference" [Title/Abstract) filter: humans.

<u>Scopus</u>

TITLE-ABS (fetal OR prenatal OR "pre-natal" OR gestation OR "post-natal" OR postnatal OR neonatal OR infant OR infancy OR baby OR maternal OR pregnancy) AND TITLE-ABS (birthweight OR "birth weight" OR "birth length" OR "head circumference") AND TITLE-ABS (mercury OR methylmercury) AND (LIMIT-TO (EXACTKEYWORD , "Human")) AND (EXCLUDE (EXACTKEYWORD , "Animals"))

<u>Embase</u>

(fetal OR prenatal OR "pre-natal" OR gestation OR "post-natal" OR postnatal OR neonatal OR infant OR infancy OR baby OR maternal OR pregnancy) .ti,ab. and (exp mercury/ OR exp methylmercury/) and (birthweight OR "birth weight" OR "birth length" OR "head circumference").ti,ab. not ((exp animal/ or nonhuman/) not exp human/)

PsycINFO

(fetal OR prenatal OR "pre-natal" OR gestation OR "post-natal" OR postnatal OR neonatal OR infant OR infancy OR baby OR maternal OR pregnancy) .ti,ab. and (exp "mercury (metal)"/) OR (methylmercury.ti,ab.) and birthweight OR "birth weight" OR "birth length" OR "head circumference").ti,ab. **Part 3:** Fields used in data extraction.

Author
Year
Study design
Country of origin
Recruitment criteria
Total study sample size

Exposure source
Timing of exposure
Mean / SD of exposure
Median / IQR of exposure
Exposure units
Exposure analysis method
Exposure analysis LoD

Outcome

Model sample size
Model estimate type
Model coefficient
Model coefficient confidence intervals
Model coefficient p-value

Adjustment set

Part 4. Quality Assessment Tool

**NIH Quality assessment tool for observational cohort and cross-sectional studies**<br>**Website:** https://www.nhlbi.nih.gov/health-topics/study-quality-assessment-tools			
Major Components	Yes	No	Other (NA, NR, CD)
1. Was the research question or objective in this paper clearly stated?			
2. Was the study population clearly specified and defined?			
3. Was the participation rate of eligible persons at least 50%?			
4. Were all the subjects selected or recruited from the same or similar populations (including the same time period)? Were inclusion and exclusion criteria for being in the study prespecified and applied uniformly to all participants?			
5. Was a sample size justification, power description, or variance and effect estimates provided?			
6. For the analyses in this paper, were the exposure(s) of interest measured prior to the outcome(s) being measured?			
7. Was the timeframe sufficient so that one could reasonably expect to see an association between exposure and outcome if it existed?			
8. For exposures that can vary in amount or level, did the study examine different levels of the exposure as related to the outcome (e.g., categories of exposure, or exposure measured as continuous variable)?			

9. Were the exposure measures (independent variables) clearly defined, valid, reliable, and implemented consistently across all study participants?			
10. Was the exposure(s) assessed more than once over time? **(1)**			
11. Were the outcome measures (dependent variables) clearly defined, valid, reliable, and implemented consistently across all study participants?			
12. Were the outcome assessors blinded to the exposure status of participants?			
13. Was loss to follow-up after baseline 20% or less?			
14. Were key potential confounding variables measured and adjusted statistically for their impact on the relationship between exposure(s) and outcome(s)? **(2)**			
15. Were multivariable models based on prior theory, with covariates selected based on theory or evidence that they may be confounders or competing exposures? **(3)**			
16. Were the results of all analyses described in the study methods reported, including results which did not meet a threshold of statistical significance? **(3)**			

(1) This question was not relevant to the type of studies included in this review. To make it more useful it was taken to also include whether the study reported quality control methods when analysing mercury concentrations.
(2) Key confounders: Maternal socio-economic status or education, fish or fatty acid intake, maternal smoking status. Based on confounders identified in:

Nagpal N, Bettiol S, Isham A, Hoang H, Crocombe L. 2017. A review of mercury exposure and health of dental personnel. Safety and Health at Work. 8, pp.1-10.
World Health Organization. 2008. Guidance for identifying populations at risk from mercury exposure. Available online: https://www.who.int/foodsafety/publications/chem/mercuryexposure.pdf

(3) These are additional criteria added to evaluate common statistical issues in perinatal research.

Part 5. Mercury exposure characteristics in included studies.

			**Mercury concentrations**					
**Study (year)**	**Country**	**Exposure**	**Mean (SD)**	**Median (IQR)**	**Units**	**Mercury analysis method**	**Limits of detection**	**Timing of exposure**
Baldewsingh et al (2020)	Suriname	Hair		3.48	µg/g	CVAAS		27 weeks gestation
Bashore et al (2014)	USA	Umbilical cord<br>Urine				ICP-MS	Urine: 0.09 µg/L<br>Cord: 0.24 µg/L	At delivery<br>6 - 9 months gestation
Bloom et al (2015)	USA	Whole blood	1.85 (2.17)	1.11	µg/L	ICP-MS		Pre-pregnancy
Ding et al (2013)	China	Umbilical cord<br>Whole blood	1.60 (0.72)<br>0.91 (0.37)	1.5<br>0.9	µg/L	CVAAS	0.3 µg/L	At delivery<br>Upon admission for delivery
Eguchi et al (2019)	Japan	Blood serum	1.0 (0.57)		ng/g	ICP-MS	0.05 ng/g	32 weeks gestation
Gustin et al (2020)	Sweden	Erythrocyte		1.5	µg/kg	ICP-MS	0.01 µg/kg	28 weeks gestation
Howe et al (2020)	USA	Urine		1.02 (1.5)	µg/L	ICP-MS		13 weeks gestation
Kim et al (2017)	South Korea, Taiwan	Umbilical cord<br>Whole blood		5.75 (4.37)<br>3.27 (2.25)	µg/L	ICP-MS	0.158 µg/L & 0.28 µg/L	At delivery<br>28-42 weeks gestation
Lee et al (2010)	South Korea	Umbilical cord<br>Whole blood	5.53*<br>3.67*<br>3.30*	5.54<br>3.85<br>3.23	µg/L	AAS	0.158 µg/L	At delivery<br>12-20 & 28-42 weeks gestation
Taylor et al (2016)	United Kingdom	Whole blood	2.07 (1.1)		µg/L	ICP-MS	0.24 µg/L	11 weeks gestation

Vigeh et al (2018)	Japan	Whole blood	6.06 (3.81)<br>4.99 (3.45)<br>4.97 (3.25)		µg/L	AAS	0.003 ng/mL	First trimester
Al-Saleh et al (2014)	Saudi Arabia	Placenta<br>Umbilical cord	0.056 (0.075)<br>3.46 (2.50)	0.032<br>3.15	µg/g dry weight<br>µg/L	AAS	0.033 µg/g dry wt.	At delivery
Arinola et al (2018)	Nigeria	Whole blood			Not reported	AAS		At delivery
Foldspang & Hansen (1990)	Greenland	Umbilical cord<br>Whole blood	21.0<br>14.9		µg/L	AAS		At delivery
Freire et al (2019)	Spain	Placenta		4.43 (17.09)	ng/g	AAS	0.016 ng/g	At delivery
Govarts et al (2016)	Belgium	Hair	0.255*	(0.28)	µg/g	HG-GC-AFS (headspace injection-gas chromatography-atomic fluorescence spectrometry(	0.00004 µg/g	At delivery
Grandjean et al (2001)	Faroe Islands	Umbilical cord	101.7*	(140.1)	nmol/l			At delivery
Guo et al (2013)	China	Child hair<br>Hair<br>Placenta<br>Umbilical cord	289.6 (392.5)<br>607.4 (182.4)<br>4.10 (2.1)<br>1.8 (1.1)	249.1 (405.3)<br>518.6 (827.7)<br>3.7 (2.5)<br>1.5 (1.8)	µg/kg<br>µg/kg<br>µg/kg<br>µg/L	AAS	Hair, placenta: 0.03 µg/kg<br>Cord: 0.3 µg/L	At delivery
Marques et al (2013)	Brazil	Child hair<br>Hair				CVAAS		At delivery
Murcia et al (2016)	Spain	Umbilical cord	8.2*		µg/L	AAS	2 µg/L	At delivery
Rahbar et al (2015)	Jamaica	Umbilical cord	4.4 (2.4)	4.0 (2.4)	µg/L	ICP-MS	0.25 µg/L	At delivery
Ramón et al (2009)	Spain	Umbilical cord	9.4*		µg/L	AAS	2 µg/L	At delivery
Tatsuta et al (2017)	Japan	Umbilical cord			ng/g	CVAAS		At delivery

Wells et al (2016)	USA	Umbilical cord	0.94*		μg/L	ICP-MS	0.48 μg/L	At delivery
van Wijngaarden et al (2014)	Republic of Seychelles	Umbilical cord	5.9 (3.9)	5.1	μg/g	CVAAS		At delivery
Yeates et al (2020)	Republic of Seychelles	Hair			μg/g	AAS		At delivery
Hong (2017)	China	Hair (THg)<br>Hair (MeHg)<br>Whole blood	0.48 (0.26)<br>0.32 (0.2)<br>1.5 (1)		μg/g<br>μg/g<br>μg/L	Hair: CV-AFS (cold vapor atomic fluorescence spectrometry)<br>Blood: CVAAS	Hair THg: 0.0095 μg/g<br>Hair MeHg: 0.0001 μg/g<br>Blood: 0.14 μg/L	At delivery

* Geometric mean

Part 6: Results reported in included studies.

A. Birth weight.

Study	n	Exposure	Units	Outcome	Estimate type	Notes	Estimate	LCI	UCI	P-value
Eguchi et al (2019)	62	Blood serum	ng/g	Birth weight	Bayesian normalized coefficient		0.04	-0.21	0.30	
Guo et al (2013)	213	Child hair	μg/kg	Birth weight	RR		0.05	-104	242	0.43
Marques et al (2013)	1433	Child hair	μg/g	Birth weight	RR		-17.74			0.18
Gustin et al (2020)	150	Erythrocyte	μg/kg	Birth weight	RR	Ery-Hg<1.0 μg /kg	58	11	105	0.02
Gustin et al (2020)	408	Erythrocyte	μg/kg	Birth weight	RR	Ery-Hg>1.0 μg /kg	-59	-115	-3	0.04
Baldewsingh et al (2020)	178	Hair	μg/g	Birth weight	RR		10.3	-4.54	25.1	0.18
Govarts et al (2016)	244	Hair	μg/g	Birth weight	Standardized RR		-19.2	-85.6	47.3	0.571
Guo et al (2013)	213	Hair	μg/kg	Birth weight	RR		0.23	-153.1	218.1	0.73
Marques et al (2013)	1433	Hair	μg/g	Birth weight	RR		1.51			0.53
Yeates et al (2020)	1111	Hair	μg/g	Birth weight	Standardized RR		0	-0.006	0.007	

Hong (2017)	383	Hair (MeHg)	Log10 µg/g	Birth weight	Standardized RR		-0.31	-0.63	0.001	
Hong (2017)	383	Hair (THg)	Log10 µg/g	Birth weight	Standardized RR		-0.41	-0.78	-0.032	
Freire et al (2019)	327	Placenta	Detected Hg	Birth weight	RR		37.6	-52.01	127	
Guo et al (2013)	213	Placenta	µg/kg	Birth weight	RR		0.08	-89.5	349	0.25
Al-Saleh et al (2014)	247	Umbilical cord	µg/L	Birth weight	Standardized RR		-0.03			<0.05
Bashore et al (2014)	64	Umbilical cord	10% increase	Birth weight	RR		4.42	-7.38	16.22	
Ding et al (2013)	258	Umbilical cord	Log_{10} µg/L	Birth weight	RR		-19.8	-309	269	0.89
Foldspang & Hansen (1990)	376	Umbilical cord	µg/L	Birth weight	RR		-4.2			0.012
Grandjean et al (2001)	182	Umbilical cord	nmol/L	Birth weight	RR					0.63
Guo et al (2013)	213	Umbilical cord	µg/L	Birth weight	RR		-0.08	-318	72.2	0.22
Kim et al (2017)	1147	Umbilical cord	doubling in Hg	Birth weight	RR		-0.02	-0.05	0.01	
Kim et al (2017)	1147	Umbilical cord	doubling in Hg >25th percentile	Birth weight	RR		-0.07	-0.12	-0.03	
Lee et al (2010)	417	Umbilical cord	Log µg/L	Birth weight	RR		-86.4	-163	-9.7	
Murcia et al (2016)	1869	Umbilical cord	Doubling in Hg	Birth weight	RR		-14.1	-42.3	14.1	0.33
Rahbar et al (2015)	96	Umbilical cord	Log µg/L	Birth weight	RR		0.13			0.20
Ramón et al (2009)	138	Umbilical cord	Quartile 2 vs 1	Birth weight	RR		-100	-200	-0.5	
Ramón et al (2009)	145	Umbilical cord	Quartile 3 vs 1	Birth weight	RR		-76.7	-179	25.8	
Ramón et al (2009)	133	Umbilical cord	Quartile 4 vs 1	Birth weight	RR		-144	-252	-35.6	

Tatsuta et al (2017)	252	Umbilical cord	Log ng/g	Birth weight	Standardized RR	Boys	-0.12			0.04
Tatsuta et al (2017)	237	Umbilical cord	Log ng/g	Birth weight	Standardized RR	Girls	-0.04			0.50
Tatsuta et al (2017)	489	Umbilical cord	Log ng/g	Birth weight	Standardized RR		-0.08			0.06
Wells et al (2016)	271	Umbilical cord	Log μg/L	Birth weight	RR		-29.6	-93.9	34.6	
van Wijngaarden et al (2014)	230	Umbilical cord	μg/g	Birth weight	RR		-7.7	-7.9	23.3	
Bashore et al (2014)	140	Urine	10% increase	Birth weight	RR		-1.23	-7.35	4.88	
Howe et al (2020)	262	Urine	1st to 3rd quartile increase	Birth weight	Standardized RR		-0.11	-0.31	0.09	
Arinola et al (2018)	67	Whole blood	Not reported	Birth weight	RR		-0.10			<0.05
Ding et al (2013)	258	Whole blood	Log_{10} μg/L	Birth weight	RR		0.48	-303	304	0.99
Foldspang & Hansen (1990)	376	Whole blood	μg/L	Birth weight	RR		-7.1			0.019
Hong (2017)	383	Whole blood	Log10 μg/L	Birth weight	Standardized RR		-0.36	-0.73	0.01	
Kim et al (2017)	1147	Whole blood	doubling in Hg	Birth weight	RR	Kilograms	-0.003	-0.03	0.02	
Kim et al (2017)	1147	Whole blood	doubling in Hg >25th percentile	Birth weight	RR	Kilograms	-0.06	-0.10	-0.01	
Taylor et al (2016)	2693	Whole blood	μg/L	Birth weight	RR		-4.15	-20.5	12.2	0.62
Taylor et al (2016)	2324	Whole blood	μg/L	Birth weight	RR	Fish eaters	-3.28	-21.0	14.5	0.72
Taylor et al (2016)	354	Whole blood	μg/L	Birth weight	RR	Non fish eaters	-57	-113	-1.49	0.044
Vigeh et al (2018)	334	Whole blood	Log μg/L	Birth weight	Standardized RR	First trimester	-0.17			0.006
Bloom et al (2015)	232	Whole blood	2nd tertile vs 1st	Birth weight	RR		146	-5.52	297	
Bloom et al (2015)	232	Whole blood	3rd tertile vs 1st	Birth weight	RR		137	-22.5	297	
Lee et al (2010)	417	Whole blood (early)	Log μg/L	Birth weight	RR		-68.6	-139	1.5	

Lee et al (2010)	417	Whole blood (late)	Log µg/L	Birth weight	RR		-65.5	-136	4.5	

B. Birth length.

**Study**	**n**	**Exposure**	**Units**	**Outcome**	**Estimate type**	**Notes**	**Estimate**	**LCI**	**UCI**	**P-value**
Guo et al (2013)	213	Child hair	µg/kg	Birth length	RR		0.04	-0.39	0.74	0.54
Gustin et al (2020)	148	Erythrocyte	µg/kg	Birth length	RR	Ery-Hg<1.0 µg /kg	0.24	0.02	0.45	0.03
Gustin et al (2020)	397	Erythrocyte	µg/kg	Birth length	RR	Ery-Hg>1.0 µg /kg	-0.29	-0.54	-0.047	0.02
Guo et al (2013)	213	Hair	µg/kg	Birth length	RR		-0.34	-0.76	0.46	0.63
Yeates et al (2020)	1092	Hair	µg/g	Birth length	Standardized RR		-0.02	-0.07	0.03	
Hong (2017)	383	Hair (MeHg)	Log10 µg/g	Birth length	Standardized RR		-0.2	-0.6	0.21	
Hong (2017)	383	Hair (THg)	Log10 µg/g	Birth length	Standardized RR		-0.28	-0.72	0.15	
Al-Saleh et al (2014)	249	Placenta	µg/g dry wt.	Birth length	Standardized RR		-0.22			<0.01
Freire et al (2019)	327	Placenta	Detected Hg	Birth length	RR		-0.47	-0.99	0.05	<0.05
Guo et al (2013)	213	Placenta	µg/kg	Birth length	RR		0.04	-0.51	0.93	0.56
Al-Saleh et al (2014)	247	Umbilical cord	µg/L	Birth length	Standardized RR		0.14			NS
Bashore et al (2014)	62	Umbilical cord	10% increase	Birth length	RR		-0.24	-10.5	9.98	
Ding et al (2013)	258	Umbilical cord	Log$_{10}$ µg/L	Birth length	RR		-0.1	-1.28	1.08	0.87
Guo et al (2013)	213	Umbilical cord	µg/L	Birth length	RR		-0.01	-0.69	0.59	0.89
Murcia et al (2016)	1869	Umbilical cord	Doubling in Hg	Birth length	RR		-0.05	-0.12	0.03	0.24
Rahbar et al (2015)	48	Umbilical cord	Log µg/L	Birth length	RR		-0.07			0.64
Ramón et al (2009)	138	Umbilical cord	Quartile 2 vs 1	Birth length	RR		-0.44	-0.89	0.01	

Ramón et al (2009)	145	Umbilical cord	Quartile 3 vs 1	Birth length	RR		0.07	-0.4	0.54	
Ramón et al (2009)	133	Umbilical cord	Quartile 4 vs 1	Birth length	RR		-0.31	-0.81	0.19	
Wells et al (2016)	271	Umbilical cord	Log µg/L	Birth length	RR		0.14	-0.17	0.45	
Bashore et al (2014)	133	Urine	10% increase	Birth length	RR		-1.74	-6.2	2.71	
Ding et al (2013)	258	Whole blood	Log$_{10}$ µg/l	Birth length	RR		0.39	-0.85	1.63	0.54
Hong (2017)	383	Whole blood	Log10 µg/L	Birth length	Standardized RR		0.035	-0.44	0.51	
Taylor et al (2016)	2345	Whole blood	µg/L	Birth length	RR		0.01	-0.07	0.09	0.74
Taylor et al (2016)	2026	Whole blood	µg/L	Birth length	RR	Fish eaters	0.01	-0.08	0.09	0.89
Taylor et al (2016)	310	Whole blood	µg/L	Birth length	RR	Non fish eaters	-0.08	-0.33	0.17	0.55
Bloom et al (2015)	231	Whole blood	2nd tertile vs 1st	Birth length	RR		0.68	-0.2	1.56	
Bloom et al (2015)	231	Whole blood	3rd tertile vs 1st	Birth length	RR		1.11	0.18	2.03	

C. Head circumference.

**Study**	**n**	**Exposure**	**Units**	**Outcome**	**Estimate type**	**Notes**	**Estimate**	**LCI**	**UCI**	**P-value**
Eguchi et al (2019)	62	Blood serum	ng/g	Head circumference	Bayesian normalized coefficient		0.03	-0.25	0.3	
Guo et al (2013)	213	Child hair	µg/kg	Head circumference	RR		-0.12	-0.82	0.04	0.09
Gustin et al (2020)	101	Erythrocyte	µg/kg	Head circumference	RR	Ery-Hg<0.76 µg /kg	0.12	-0.04	0.29	0.15
Gustin et al (2020)	444	Erythrocyte	µg/kg	Head circumference	RR	Ery-Hg>0.76 µg /kg	-0.14	-0.29	0.01	0.06

Guo et al (2013)	213	Hair	µg/kg	Head circumference	RR		-0.06	-0.65	0.28	0.43
Yeates et al (2020)	1091	Hair	µg/g	Head circumference	Standardized RR		0.01	-0.014	0.04	
Hong (2017)	383	Hair (MeHg)	Log10 µg/g	Head circumference	Standardized RR		-0.06	-0.15	0.04	
Hong (2017)	383	Hair (THg)	Log10 µg/g	Head circumference	Standardized RR		-0.08	-0.19	0.03	
Freire et al (2019)	293	Placenta	Detected Hg	Head circumference	RR		-0.02	-0.33	0.29	
Guo et al (2013)	213	Placenta	µg/kg	Head circumference	RR		0.03	-0.41	0.69	0.62
Al-Saleh et al (2014)	247	Umbilical cord	µg/L	Head circumference	Standardized RR		0.03			NS
Bashore et al (2014)	64	Umbilical cord	10% increase	Head circumference	RR		61.2	-66.2	188.3	
Ding et al (2013)	258	Umbilical cord	Log_{10} µg/L	Head circumference	RR		-0.27	-2.37	1.82	0.8
Guo et al (2013)	213	Umbilical cord	µg/L	Head circumference	RR		0.02	-0.44	0.54	0.85
Murcia et al (2016)	1869	Umbilical cord	Doubling in Hg	Head circumference	RR		-0.05	-0.11	0.005	0.07
Rahbar et al (2015)	55	Umbilical cord	Log µg/L	Head circumference	RR		-0.13			0.35
Wells et al (2016)	271	Umbilical cord	Log µg/L	Head circumference	RR		-0.16	-0.37	0.04	
Bashore et al (2014)	137	Urine	10% increase	Head circumference	RR		3.63	-66.8	74.1	
Ding et al (2013)	258	Whole blood	Log_{10} µg/l	Head circumference	RR		1.49	-0.71	3.68	0.18
Hong (2017)	383	Whole blood	Log10 µg/L	Head circumference	Standardized RR		-0.13	-0.24	-0.02	

Taylor et al (2016)	2376	Whole blood	µg/L	Head circumference	RR		0.01	-0.04	0.05	0.85
Taylor et al (2016)	2055	Whole blood	µg/L	Head circumference	RR	Fish eaters	0	-0.05	0.05	0.96
Taylor et al (2016)	311	Whole blood	µg/L	Head circumference	RR	Non fish eaters	-0.05	-0.24	0.15	0.63
Bloom et al (2015)	182	Whole blood	2nd tertile vs 1st	Head circumference	RR		-0.12	-0.8	0.57	
Bloom et al (2015)	182	Whole blood	3rd tertile vs 1st	Head circumference	RR		0.11	-0.61	0.84	

Part 7. Data used in albatross plots.
Each study was included once per outcome and mercury exposure type (e.g., hair, placenta).
In cases where a study reported more than one primary result per outcome and mercury exposure type (e.g., Bloom et al, 2015, reported both 2nd tertile vs 1st tertile maternal blood Hg, and 3rd tertile vs 1st tertile maternal blood Hg) the following priorities were used to select a single estimate:
1. The broadest group (e.g., estimates for boys, girls, and both combined; select estimate for both combined).
2. The widest ranging categories (e.g., estimates for 3rd vs 1st Hg tertile, and 2nd vs 1st Hg tertile; select estimate for 3rd vs 1st Hg tertile)

Study	*n*	Exposure	category	beta	*p*
Al-Saleh et al (2014)	247	Umbilical cord	Birth weight	-0.032	0.0049
Arinola et al (2018)	67	Whole blood	Birth weight	-0.10	0.0049
Baldewsingh et al (2020)	178	Hair	Birth weight	10.3	0.18
Bashore et al (2014)	140	Urine	Birth weight	-1.23	0.71
Bashore et al (2014)	64	Umbilical cord	Birth weight	4.42	0.47
Bloom et al (2015)	232	Whole blood	Birth weight	137	0.09
Ding et al (2013)	258	Umbilical cord	Birth weight	-19.8	0.89
Ding et al (2013)	258	Whole blood	Birth weight	0.48	0.99
Eguchi et al (2019)	62	Blood serum	Birth weight	0.04	0.74
Foldspang & Hansen (1990)	376	Whole blood	Birth weight	-7.1	0.02
Foldspang & Hansen (1990)	376	Umbilical cord	Birth weight	-4.2	0.01
Freire et al (2019)	327	Placenta	Birth weight	37.6	0.42
Govarts et al (2016)	244	Hair	Birth weight	-19.2	0.57
Guo et al (2013)	213	Umbilical cord	Birth weight	-0.08	0.22
Guo et al (2013)	213	Child hair	Birth weight	0.05	0.43
Guo et al (2013)	213	Placenta	Birth weight	0.08	0.25
Guo et al (2013)	213	Hair	Birth weight	0.23	0.73
Gustin et al (2020)	408	Erythrocyte	Birth weight	-59	0.04
Hong (2017)	383	Hair	Birth weight	-0.41	0.03
Hong (2017)	383	Whole blood	Birth weight	-0.36	0.06
Howe et al (2020)	262	Urine	Birth weight	-0.11	0.28
Kim et al (2017)	1147	Umbilical cord	Birth weight	-0.02	0.14
Kim et al (2017)	1147	Whole blood	Birth weight	-0.003	0.84
Lee et al (2010)	417	Umbilical cord	Birth weight	-86.4	0.03
Lee et al (2010)	417	Whole blood	Birth weight	-68.6	0.05
Marques et al (2013)	1433	Child hair	Birth weight	-17.7	0.18
Marques et al (2013)	1433	Hair	Birth weight	1.51	0.53
Murcia et al (2016)	1869	Umbilical cord	Birth weight	-14.1	0.33
Rahbar et al (2015)	96	Umbilical cord	Birth weight	0.13	0.20
Ramon et al (2009)	133	Umbilical cord	Birth weight	-144	0.01
Tatsuta et al (2017)	489	Umbilical cord	Birth weight	-0.08	0.06
Taylor et al (2016)	2693	Whole blood	Birth weight	-4.15	0.62
Vigeh et al (2018)	334	Whole blood	Birth weight	-0.17	0.006
Wells et al (2016)	271	Umbilical cord	Birth weight	-29.6	0.37

van Wijngaarden et al (2014)	230	Umbilical cord	Birth weight	-7.7	0.34
Yeates et al (2020)	1111	Hair	Birth weight	0.0001	1

**Study**	***n***	**Exposure**	**category**	**beta**	***p***
Al-Saleh et al (2014)	249	Placenta	Birth length	-0.22	0.009
Bashore et al (2014)	133	Urine	Birth length	-1.74	0.45
Bashore et al (2014)	62	Umbilical cord	Birth length	-0.24	0.97
Bloom et al (2015)	231	Whole blood	Birth length	1.11	0.02
Ding et al (2013)	258	Umbilical cord	Birth length	-0.10	0.87
Ding et al (2013)	258	Whole blood	Birth length	0.39	0.54
Freire et al (2019)	327	Placenta	Birth length	-0.47	0.0049
Guo et al (2013)	213	Hair	Birth length	-0.34	0.63
Guo et al (2013)	213	Umbilical cord	Birth length	-0.01	0.89
Guo et al (2013)	213	Child hair	Birth length	0.04	0.54
Guo et al (2013)	213	Placenta	Birth length	0.04	0.56
Gustin et al (2020)	397	Erythrocyte	Birth length	-0.29	0.02
Hong (2017)	383	Hair	Birth length	-0.28	0.21
Hong (2017)	383	Whole blood	Birth length	0.04	0.89
Murcia et al (2016)	1869	Umbilical cord	Birth length	-0.05	0.24
Rahbar et al (2015)	48	Umbilical cord	Birth length	-0.07	0.64
Ramon et al (2009)	133	Umbilical cord	Birth length	-0.31	0.23
Taylor et al (2016)	2345	Whole blood	Birth length	0.01	0.74
Wells et al (2016)	271	Umbilical cord	Birth length	0.14	0.38
Yeates et al (2020)	1092	Hair	Birth length	-0.02	0.06

**Study**	***n***	**Exposure**	**category**	**beta**	***p***
Bashore et al (2014)	137	Urine	Head circumference	3.63	0.93
Bashore et al (2014)	64	Umbilical cord	Head circumference	61.2	0.35
Bloom et al (2015)	182	Whole blood	Head circumference	0.11	0.78
Ding et al (2013)	258	Umbilical cord	Head circumference	-0.27	0.80
Ding et al (2013)	258	Whole blood	Head circumference	1.49	0.18
Eguchi et al (2019)	62	Blood serum	Head circumference	0.03	0.85
Freire et al (2019)	293	Placenta	Head circumference	-0.02	0.91
Guo et al (2013)	213	Child hair	Head circumference	-0.12	0.09
Guo et al (2013)	213	Hair	Head circumference	-0.06	0.43
Guo et al (2013)	213	Umbilical cord	Head circumference	0.02	0.85
Guo et al (2013)	213	Placenta	Head circumference	0.03	0.62
Gustin et al (2020)	444	Erythrocyte	Head circumference	-0.14	0.06
Hong (2017)	383	Whole blood	Head circumference	-0.13	0.02
Hong (2017)	383	Hair	Head circumference	-0.081	0.15
Murcia et al (2016)	1869	Umbilical cord	Head circumference	-0.052	0.07
Rahbar et al (2015)	55	Umbilical cord	Head circumference	-0.13	0.35
Taylor et al (2016)	2055	Whole blood	Head circumference	0.0001	0.96
Wells et al (2016)	271	Umbilical cord	Head circumference	-0.16	0.13

Yeates et al (2020)	1091	Hair	Head circumference	0.01	0.43

Part 8. Results of NIH Quality Assessment.

Study (year)	1	2	3	4	5	6	7	8	9	10	11	12	13	14	15	16	Total
Baldewsingh et al (2020)	1	1	NR	1	0	1	1	1	1	0	1	1	1	0	1	1	12
Bashore et al (2014)	1	1	NR	1	1	1	1	1	1	1	1	1	1	0	1	1	14
Bloom et al (2015)	1	1	NR	1	1	1	1	1	1	1	1	1	0	0	1	1	13
Ding et al (2013)	1	1	1	1	1	1	1	1	1	1	1	1	0	0	1	1	13
Eguchi et al (2019)	1	0	NR	NR	0	1	1	1	1	1	1	1	0	0	1	1	10
Gustin et al (2020)	1	1	NR	0	1	1	1	1	1	1	1	1	1	0	0	1	12
Howe et al (2020)	1	1	NR	1	1	1	1	1	1	1	1	1	NR	0	1	1	13
Kim et al (2017)	1	1	NR	0	1	1	1	1	1	1	1	1	0	0	0	1	10
Lee et al (2010)	1	1	NR	1	1	1	1	1	1	1	1	1	0	0	1	1	12
Taylor et al (2016)	1	1	NR	1	0	1	1	1	1	1	1	1	NR	1	1	1	13
Vigeh et al (2018)	1	1	1	1	1	1	1	1	1	1	1	1	1	1	1	1	16
Al-Saleh et al (2014)	1	1	NR	1	0	0	1	1	1	1	1	1	NR	1	0	0	10
Arinola et al (2018)	1	1	NR	1	0	0	0	1	0	1	0	1	NR	0	1	1	8
Foldspang & Hansen (1990)	1	1	1	1	1	0	1	1	1	1	1	1	NA	1	1	1	13
Freire et al (2019)	1	1	NR	1	1	0	1	1	1	1	1	1	NA	0	1	1	12
Govarts et al (2016)	1	1	NR	1	0	0	1	1	1	1	1	1	NA	0	1	1	11
Grandjean et al (2001)	1	1	1	1	1	0	1	1	1	0	1	1	NA	0	0	0	10
Guo et al (2013)	1	1	1	1	1	0	1	1	1	1	1	1	NA	0	1	1	13
Marques et al (2013)	1	1	1	1	1	0	1	1	1	0	1	1	NA	0	1	1	12
Murcia et al (2016)	1	1	1	1	1	0	1	1	1	0	1	1	NA	1	1	1	13
Rahbar et al (2015)	1	1	NR	0	0	0	1	1	1	0	1	1	NA	0	1	1	9
Ramon et al (2009)	1	1	1	0	0	0	1	1	1	1	1	1	NA	1	1	1	12
Tatsuta et al (2017)	1	1	1	1	1	0	1	1	1	1	1	1	NA	1	1	1	14
Wells et al (2016)	1	1	1	1	1	0	1	1	1	1	1	1	NA	1	1	1	14
van Wijngaarden et al (2014)	1	1	NR	1	1	0	1	1	1	0	1	1	NA	1	1	1	12

Yeates et al (2020)	1	1	NR	1	0	0	1	1	1	0	1	1	NA	1	1	1	11
Hong (2017)	1	1	0	0	0	1	1	1	1	1	1	1	1	1	1	1	13

NR = not reported
NA = not applicable

Part 9. Results reported in high quality studies and albatross plots.
High quality: Studies which met 12+ QA criteria and met criteria 14 of the quality assessment tool: "Were key potential confounding variables measured and adjusted statistically for their impact on the relationship between exposure(s) and outcome(s)?"

**Study**	***n***	**Exposure**	**Units**	**Outcome**	**Estimate type**	**Notes**	**Estimate**	**LCI**	**UCI**	***p*-value**
Foldspang & Hansen (1990)	376	Umbilical cord	μg/l	Birth weight	RR		-4.2			0.012
Foldspang & Hansen (1990)	376	Whole blood	μg/l	Birth weight	RR		-7.1			0.019
Hong (2017)	383	Hair (MeHg)	Log10 μg/g	Birth weight	Standardized RR		-0.31	-0.63	0.001	
Hong (2017)	383	Hair (THg)	Log10 μg/g	Birth weight	Standardized RR		-0.41	-0.78	-0.03	
Hong (2017)	383	Whole blood	Log10 μg/L	Birth weight	Standardized RR		-0.36	-0.73	0.01	
Murcia et al (2016)	1869	Umbilical cord	Doubling in Hg	Birth weight	RR		-14.1	-42.3	14.1	0.33
Ramon et al (2009)	133	Umbilical cord	Quartile 4 vs 1	Birth weight	RR		-144	-252	-35.6	
Ramon et al (2009)	138	Umbilical cord	Quartile 2 vs 1	Birth weight	RR		-100	-200	-0.5	
Ramon et al (2009)	145	Umbilical cord	Quartile 3 vs 1	Birth weight	RR		-76.7	-179	25.8	
Tatsuta et al (2017)	237	Umbilical cord	Log ng/g	Birth weight	RR	Girls	-0.04			0.50

Tatsuta et al (2017)	252	Umbilical cord	Log ng/g	Birth weight	RR	Boys	-0.12			0.04
Tatsuta et al (2017)	489	Umbilical cord	Log ng/g	Birth weight	RR		-0.08			0.06
Taylor et al (2016)	354	Whole blood	µg/L	Birth weight	RR	Non fish eaters	-57	-113	-1.49	0.04
Taylor et al (2016)	2324	Whole blood	µg/L	Birth weight	RR	Fish eaters	-3.28	-21.0	14.5	0.72
Taylor et al (2016)	2693	Whole blood	µg/L	Birth weight	RR		-4.15	-20.5	12.2	0.62
Vigeh et al (2018)	334	Whole blood	Log µg/L	Birth weight	Standardized RR		-0.17			0.006
Wells et al (2016)	271	Umbilical cord	Log µg/L	Birth weight	RR		-29.6	-93.9	34.6	
Wijngaarden et al (2014)	230	Umbilical cord	ppm	Birth weight	RR		-7.7	-7.9	23.3	
Yeates et al (2020)	1111	Hair	ppm	Birth weight	Standardized RR		0	-0.006	0.007	
Hong (2017)	383	Hair (MeHg)	Log10 µg/g	Birth length	Standardized RR		-0.2	-0.6	0.21	
Hong (2017)	383	Hair (THg)	Log10 µg/g	Birth length	Standardized RR		-0.28	-0.72	0.15	
Hong (2017)	383	Whole blood	Log10 µg/L	Birth length	Standardized RR		0.035	-0.44	0.51	
Murcia et al (2016)	1869	Umbilical cord	Doubling in Hg	Birth length	RR		-0.05	-0.12	0.03	0.24
Ramon et al (2009)	138	Umbilical cord	Quartile 2 vs 1	Birth length	RR		-0.44	-0.89	0.01	
Ramon et al (2009)	145	Umbilical cord	Quartile 3 vs 1	Birth length	RR		0.07	-0.4	0.54	
Ramon et al (2009)	133	Umbilical cord	Quartile 4 vs 1	Birth length	RR		-0.31	-0.81	0.19	

Taylor et al (2016)	2345	Whole blood	µg/L	Birth length	RR		0.01	-0.07	0.09	0.74
Taylor et al (2016)	2026	Whole blood	µg/L	Birth length	RR	Fish eaters	0.01	-0.08	0.09	0.89
Taylor et al (2016)	310	Whole blood	µg/L	Birth length	RR	Non fish eaters	-0.08	-0.33	0.17	0.55
Wells et al (2016)	271	Umbilical cord	Log µg/L	Birth length	RR		0.14	-0.17	0.45	
Hong (2017)	383	Hair (MeHg)	Log10 µg/g	Head circumference	Standardized RR		-0.06	-0.15	0.04	
Hong (2017)	383	Hair (THg)	Log10 µg/g	Head circumference	Standardized RR		-0.08	-0.19	0.03	
Hong (2017)	383	Whole blood	Log10 µg/L	Head circumference	Standardized RR		-0.13	-0.24	-0.08	
Murcia et al (2016)	1869	Umbilical cord	Doubling in Hg	Head circumference	RR		-0.05	-0.11	0.005	0.07
Taylor et al (2016)	2376	Whole blood	µg/L	Head circumference	RR		0.01	-0.04	0.05	0.85
Taylor et al (2016)	2055	Whole blood	µg/L	Head circumference	RR	Fish eaters	0	-0.05	0.05	0.96
Taylor et al (2016)	311	Whole blood	µg/L	Head circumference	RR	Non fish eaters	-0.05	-0.24	0.15	0.63
Wells et al (2016)	271	Umbilical cord	Log µg/L	Head circumference	RR		-0.16	-0.37	0.04	

Deduplicated data used in albatross plots:

Study	n	Exposure	category	direction	p
Foldspang & Hansen (1990)	376	Whole blood	Birth weight	-7.1	0.019
Foldspang & Hansen (1990)	376	Umbilical cord	Birth weight	-4.2	0.012
Hong (2017)	383	Hair	Birth weight	-0.41	0.03
Hong (2017)	383	Whole blood	Birth weight	-0.36	0.06
Howe et al (2020)	262	Urine	Birth weight	-0.11	0.28
Murcia et al (2016)	1869	Umbilical cord	Birth weight	-14.1	0.33
Ramon et al (2009)	133	Umbilical cord	Birth weight	-144	0.009
Tatsuta et al (2017)	489	Umbilical cord	Birth weight	-0.08	0.061
Taylor et al (2016)	2693	Whole blood	Birth weight	-4.15	0.62
Vigeh et al (2018)	334	Whole blood	Birth weight	-0.17	0.006
Wells et al (2016)	271	Umbilical cord	Birth weight	-29.6	0.37
Wijngaarden et al (2014)	230	Umbilical cord	Birth weight	-7.7	0.34

Study	n	Exposure	category	direction	p
Hong (2017)	383	Hair	Birth length	-0.28	0.21
Hong (2017)	383	Whole blood	Birth length	0.04	0.89
Murcia et al (2016)	1869	Umbilical cord	Birth length	-0.05	0.24
Rahbar et al (2015)	48	Umbilical cord	Birth length	-0.07	0.64
Ramon et al (2009)	133	Umbilical cord	Birth length	-0.31	0.23
Taylor et al (2016)	2345	Whole blood	Birth length	0.01	0.74
Wells et al (2016)	271	Umbilical cord	Birth length	0.14	0.38

Study	n	Exposure	category	direction	p
Hong (2017)	383	Whole blood	Head circumference	-0.13	0.02
Hong (2017)	383	Hair	Head circumference	-0.08	0.15
Murcia et al (2016)	1869	Umbilical cord	Head circumference	-0.05	0.07
Rahbar et al (2015)	55	Umbilical cord	Head circumference	-0.13	0.35
Taylor et al (2016)	2055	Whole blood	Head circumference	0.0001	0.96
Wells et al (2016)	271	Umbilical cord	Head circumference	-0.16	0.13

Albatross plots:

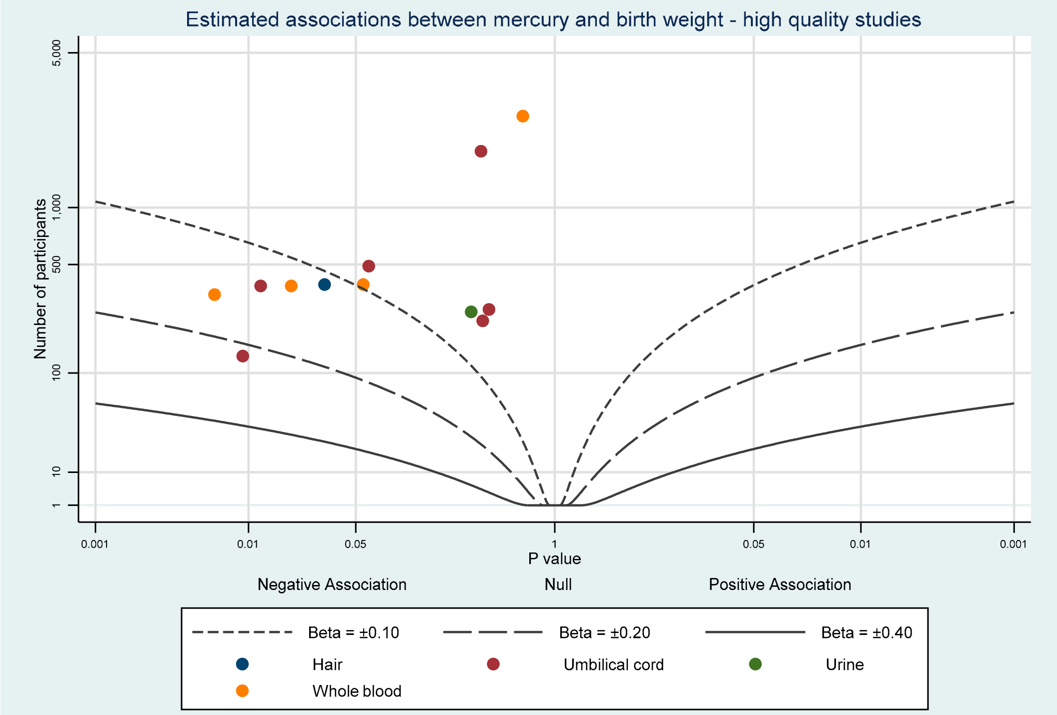
Estimated associations between mercury and birth weight - high quality studies
Number of participants
5,000
1,000
500
100
10
1
0.001
0.01
0.05
1
0.05
0.01
0.001
P value
Negative Association
Null
Positive Association
Beta = ±0.10
Beta = ±0.20
Beta = ±0.40
Hair
Umbilical cord
Urine
Whole blood

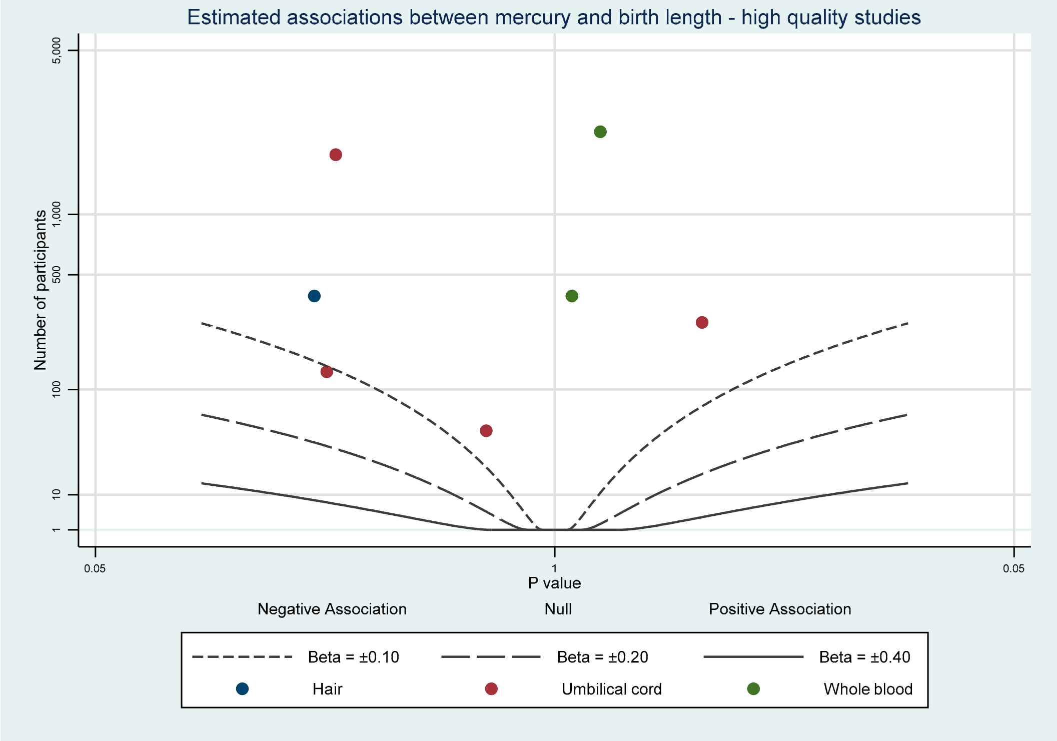
Estimated associations between mercury and birth length - high quality studies
Number of participants
5,000
1,000
500
100
10
1
0.05
1
0.05
P value
Negative Association
Null
Positive Association
Beta = ±0.10
Beta = ±0.20
Beta = ±0.40
Hair
Umbilical cord
Whole blood

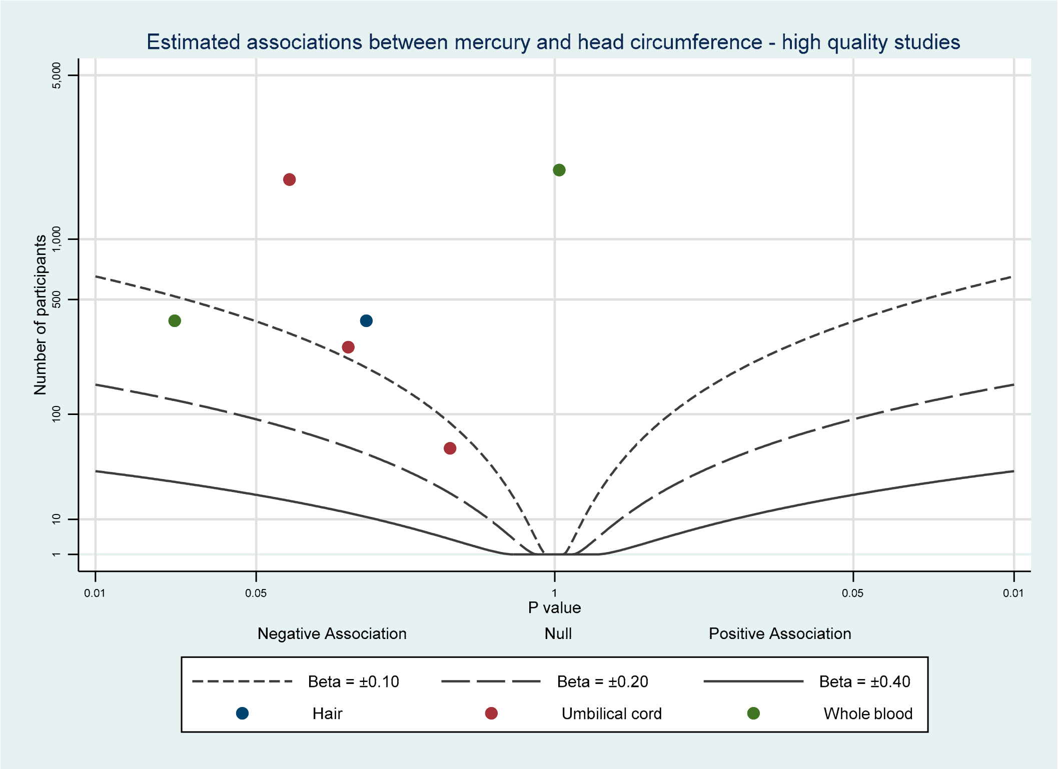

Part 10. Publication bias analysis.

For each outcome and biological matrix where there are an adequate number of results (>3), A regression test for funnel plot asymmetry (Egger test) was ran to assess potential publication bias. All results included in albatross plots were included in the Egger tests (Data taken from Supplementary File Part 7). Analysis conducted using R package *metafor*.

Birth weight & whole blood ($n = 9$): $p = 0.97$

Birth weigh & umbilical cord ($n = 13$): $p = 0.20$

Birth length & whole blood ($n = 4$): $p = 0.06$

Birth length & umbilical cord ($n = 7$): $p = 0.90$

Head circumference & whole blood ($n = 4$): $p = 0.94$

Head circumference & umbilical cord ($n = 6$): $p = 0.97$
